# Comparison of Artificial Intelligence Models Using CT Radiomics for Predicting Post-Vertebral Augmentation Residual Back Pain in Osteoporotic Vertebral Compression Fractures

**DOI:** 10.7150/ijms.114419

**Published:** 2025-07-11

**Authors:** Chen Ge, Changwei Li, Yaoqing Zhu, Chonglin Yang, Xiangyang Xu

**Affiliations:** 1 Department of Orthopaedics, Ruijin Hospital, Shanghai Jiao Tong University School of Medicine, Shanghai, China.; 2 Department of Orthopaedics, Shanghai Key Laboratory for Prevention and Treatment of Bone and Joint Diseases, Shanghai Institute of Traumatology and Orthopedics, Ruijin Hospital, Shanghai Jiao Tong University School of Medicine, Shanghai, China.

**Keywords:** osteoporotic vertebral compression fracture, residual back pain, radiomics, artificial intelligence, risk prediction, vertebral augmentation

## Abstract

**Background**: Residual back pain (RBP) following vertebral augmentation (VA) represents a significant challenge in managing osteoporotic vertebral compression fractures (OVCFs). While conventional predictive models have shown moderate accuracy, their preoperative risk stratification capabilities remain suboptimal. CT-based radiomics has demonstrated success in vertebral fracture assessment, yet its integration with artificial intelligence (AI) for predicting RBP remains unexplored.

**Objective**: This study aims to identify the optimal AI model for predicting RBP by systematically comparing multiple algorithms that integrate CT radiomics features with clinical parameters, with the goal of enabling preoperative risk stratification for improved surgical decision-making.

**Methods**: This prospective study enrolled patients who underwent VA for OVCFs. Potential predictors were identified through clinical variable analysis. Radiomics features were extracted from preoperative CT images using standardized vertebral segmentation protocols. The study population was divided into training and testing cohorts at a ratio of 7:3. Five AI models were constructed through integration of clinical predictors and radiomics features. Model performance evaluation was conducted in the independent testing cohort through discrimination, calibration, and clinical utility analyses. The predictive mechanisms of the optimal model were interpreted through feature importance analysis.

**Results**: Among 856 enrolled patients, RBP developed in 102 cases (11.9%). TabNet exhibited optimal performance metrics (AUROC: 0.927, Recall: 0.833) among all evaluated algorithms. Feature importance analysis revealed intravertebral vacuum cleft and bone mineral density as principal clinical predictors, complemented by wavelet-based texture parameters and quantitative intensity metrics. Ablation experiments demonstrated that clinical parameters were critical for false-positive reduction, while radiomics features enhanced specificity in non-RBP identification. The model maintained consistent clinical utility across varying threshold probabilities.

**Conclusion:** The integration of clinical parameters and CT-based radiomics through a deep learning framework enabled accurate preoperative prediction of RBP.

## Introduction

Osteoporotic vertebral compression fractures (OVCFs) represent a significant clinical challenge, particularly among aging populations, leading to chronic pain, functional impairment, and reduced quality of life 1, 2. Given the limited efficacy of conservative management, vertebral augmentation (VA) procedures have been established as the primary surgical intervention for symptomatic OVCFs 3. However, despite the widespread implementation of these procedures, a considerable proportion of patients continue to experience residual back pain (RBP) postoperatively, with reported incidence rates ranging from 9% to 35% 4. This persistent pain not only undermines patient confidence in surgical outcomes but also necessitates prolonged rehabilitation, thereby amplifying the burden on healthcare systems 5, 6.

Current investigations have identified multiple risk factors associated with post-operative RBP, including reduced bone mineral density (BMD), presence of intravertebral vacuum cleft (IVC), paravertebral muscle degeneration, inadequate cement distribution, and thoracolumbar fascia (TLF) injury 7, 8. Despite these advances in understanding the contributory factors, current predictive models based on conventional clinical and radiological parameters, while achieving moderate predictive accuracy, still face challenges in reliably identifying high-risk patients preoperatively, highlighting an unmet need for more robust preoperative risk stratification tools 5.

Recent advances in radiomics have demonstrated substantial potential in orthopedic research through the extraction of quantitative features from medical images 9, 10. Radiomics analysis extends beyond conventional imaging interpretation by detecting complex spatial and textural patterns within computed tomography (CT) scans, thereby revealing pathophysiological characteristics that cannot be identified through visual assessment alone 11. Multiple studies have documented the successful application of CT-based radiomics in VCFs, with validated results in several domains: the differentiation between benign and malignant fractures 12, determination of fracture acuity 13, and prediction of secondary fracture occurrence 14. However, the application of radiomics for predicting postoperative RBP remains to be investigated.

While our preliminary research has established the feasibility of a radiomics-based nomogram for RBP prediction 15, this initial model was limited by insufficient capacity to process extensive radiomic datasets, detect complex nonlinear relationships, and perform optimal feature selection. The rapid evolution of artificial intelligence (AI) technology now enables the implementation of sophisticated algorithms to analyze comprehensive radiomics and clinical parameters 16, 17. Therefore, this study aims to perform a systematic comparison of multiple AI models that incorporate CT radiomics features and clinical variables for postoperative RBP risk prediction. By systematically evaluating the performance metrics of different algorithms in processing these high-dimensional data, we seek to identify the optimal predictive model for preoperative risk stratification. This comparative analysis will not only advance our understanding of the relative strengths of different AI approaches but also establish a more reliable tool for identifying high-risk patients preoperatively, ultimately facilitating personalized treatment strategies and improved patient outcomes.

## Materials and Methods

This prospective cohort study was conducted in accordance with the Declaration of Helsinki and received approval from the Ethics Committee of Ruijin Hospital, Shanghai Jiao Tong University School of Medicine (Approval No: 2013-60). Written informed consent was obtained from all participants prior to enrollment, encompassing the use of clinical data and imaging materials for research purposes. Patient privacy was protected through comprehensive data anonymization protocols, with all personal identifiers removed from clinical records and imaging data before analysis. The study protocol and data management procedures were designed to ensure rigorous adherence to patient confidentiality standards throughout the investigation and subsequent reporting phases.

### Study design and patient selection

We evaluated patients who underwent unilateral percutaneous kyphoplasty (PKP) for OVCFs at our institution between January 2015 and January 2024. Based on Power Analysis and Sample Size (PASS) software calculations, assuming an RBP rate of 15%, a clinically significant difference of 25%, a significance level of 0.05, and a statistical power of 0.90, the minimum required sample size was determined to be 158 patients. Among 1,253 consecutive patients screened, those meeting the following inclusion criteria were selected: (1) age ≥ 55 years; (2) primary PKP intervention without previous VA; (3) single-level vertebral fracture located between T4 and L5; (4) MRI confirmation of acute fracture; (5) documented osteoporosis (T-score ≤ -2.5) on dual-energy X-ray absorptiometry; and (6) significant back pain (Visual Analog Scale [VAS] score ≥ 6) with associated functional limitations. Among patients meeting all inclusion criteria, subsequent exclusion was applied for the following conditions: (1) concurrent spinal pathologies (malignancy, active infection of vertebral body or adjacent tissues, previous surgical intervention); (2) posterior column involvement with spinal canal compromise; (3) severe cardiopulmonary disease contraindicating surgical intervention; (4) newly developed vertebral fractures within three months after surgery; and (5) insufficient follow-up data.

Standard PKP procedures were performed according to established surgical protocols through a unilateral transpedicular approach under fluoroscopic guidance. The procedure was terminated if cement extravasation was observed. Postoperatively, patients were monitored for 12 months with standardized follow-up assessments. Early mobilization was encouraged from postoperative day one, and standardized anti-osteoporotic therapy was initiated, including calcium and vitamin D supplementation combined with antiresorptive or anabolic agents based on individual risk profiles. Pain intensity was systematically evaluated using VAS at four predetermined time points: postoperative days 1, 3, 7, and 30. To minimize the confounding effect of analgesics, all VAS assessments were conducted after a standardized 12-hour washout period from short-acting analgesic medications. Based on established clinical experience and comprehensive literature analysis 18-20, RBP was defined as VAS score ≥ 4 at both postoperative day 3 and day 30, as no consensus guidelines currently exist for this clinical entity. Patients meeting these criteria were assigned to the RBP group, with the remainder constituting the control group.

### Potential clinical predictors for RBP

Clinical variables were analyzed between RBP and control groups. Preoperative baseline data included patient demographics, medical history, and BMD measurements. Fracture characteristics were documented, and functional status was evaluated using the VAS score for pain assessment and Oswestry Disability Index (ODI) for disability quantification. Preoperative imaging parameters were analyzed, including vertebral height reduction ratio, segmental Cobb angle (the angle between the superior and inferior endplate of the fractured level), presence of IVC, and TLF integrity. Statistical analysis was performed to identify potential clinical predictors, with parameters demonstrating significance at *P* < 0.1 selected for subsequent analysis.

### Image acquisition and processing

All preoperative CT examinations were performed on a 64-detector CT system (Aquilion Prime Model TSX-303A, Toshiba Medical Systems Corp., Tokyo, Japan). Standard imaging protocols were implemented with the following technical parameters: tube potential of 120-130 kV, automatic tube current modulation with a target noise index of 25, gantry rotation time of 0.5-0.75 seconds, and reconstruction matrix of 512 × 512 pixels. Image acquisition consisted of contiguous axial sections at 3-mm slice thickness, with subsequent multiplanar reformations in sagittal and coronal planes generated using dedicated postprocessing software. All imaging data were archived in Digital Imaging and Communications in Medicine (DICOM) format to maintain standardized image quality and ensure compatibility with radiomics analysis platforms.

### Image segmentation and feature extraction

**Figure 1:**
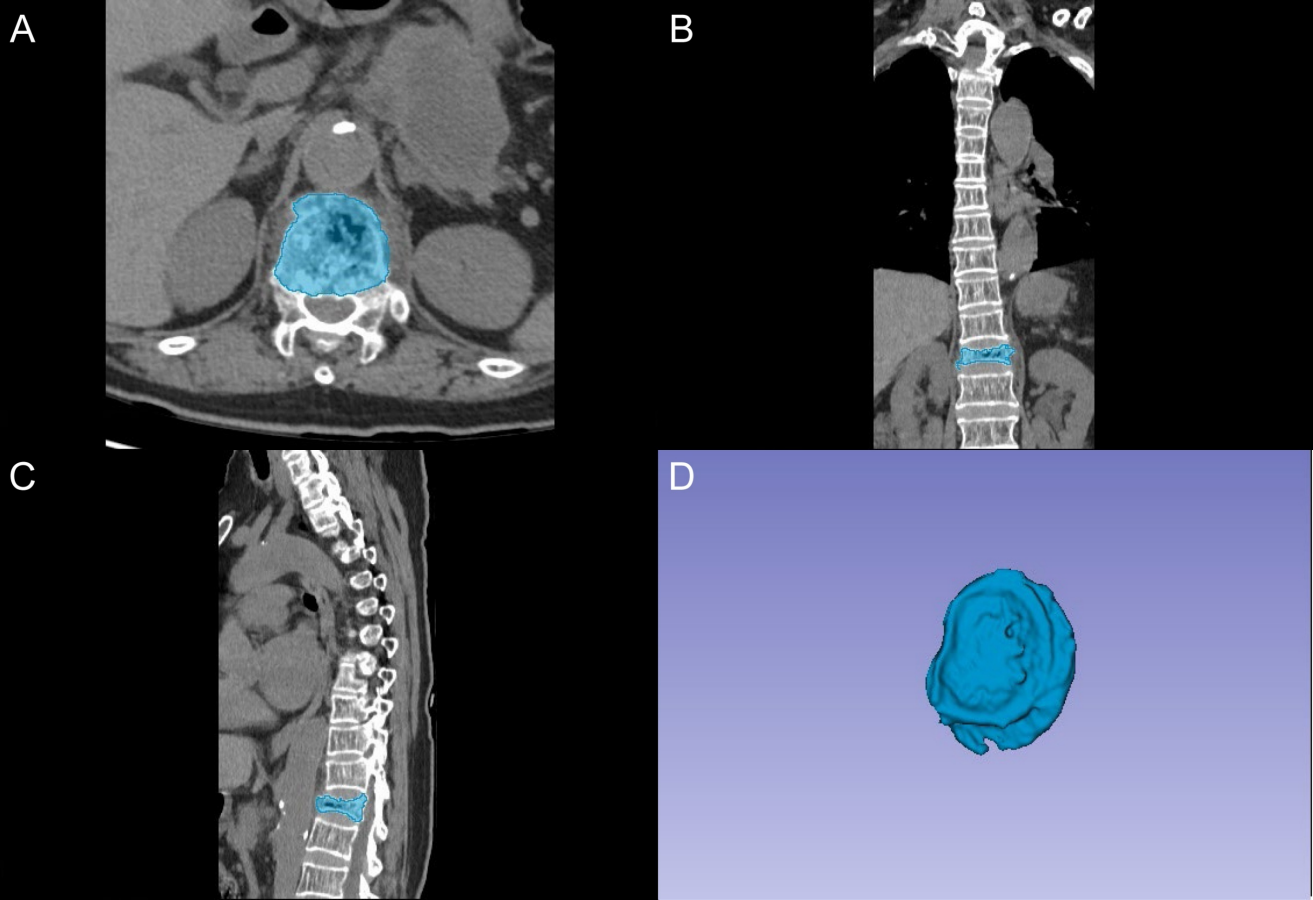
Vertebral segmentation protocol and three-dimensional reconstruction. A: Axial CT image showing semi-automatic segmentation of the vertebral body (blue). B: Coronal reconstruction demonstrating the segmented vertebral region. C: Sagittal view of the segmented vertebral body. D: Three-dimensional reconstruction of the segmented vertebral volume of interest.

The segmentation of vertebral bodies was conducted using 3D Slicer software (version 5.0.2) by two fellowship-trained radiologists with 8 and 10 years of experience in spinal imaging, who were blinded to clinical outcomes. A standardized segmentation protocol was implemented, as demonstrated in **Figure 1**. The process began with semi-automatic delineation of vertebral margins utilizing density-based thresholding to distinguish bone from adjacent soft tissues. Manual adjustments were subsequently performed to optimize boundary definition, particularly at regions of complex anatomical interfaces. The final region of interest (ROI) encompassed the complete vertebral body, including both the cancellous core and cortical shell.

Radiomics features were extracted using PyRadiomics (version 3.0) with standardized preprocessing configurations. The preprocessing parameters were systematically defined: voxel resampling to isotropic spacing (1 × 1 × 1 mm), intensity normalization with a scale factor of 100, outlier removal at 3 standard deviations, and gray-level discretization with a binning width of 25 Hounsfield units. The feature extraction pipeline was specifically configured to preserve the physical significance of CT values, implementing selected image transformations: original images, Laplacian of Gaussian (LoG) with three sigma levels (1.0, 3.0, 5.0), wavelet decomposition, and gradient maps. For each transformed image, comprehensive feature sets were computed, including shape-based metrics, first-order statistics, and higher-order textural features derived from gray-level matrices (co-occurrence, run length, size zone, dependence, and neighboring gray tone difference). Basic mask validation and correction procedures were applied to ensure segmentation integrity.

### Feature preprocessing and selection

Prior to analysis, radiomics features underwent adaptive standardization based on their statistical distributions. Features demonstrating approximately normal distributions (skewness < 2, kurtosis < 7) were processed using standard z-score normalization to achieve a mean of zero and standard deviation of one. For features with non-normal distributions or substantial outliers (>10%), robust scaling was applied, centering the data at a median of zero with an interquartile range of one, thereby minimizing the influence of extreme values.

The endpoint variable was defined as a binary outcome, with the absence or presence of RBP encoded as 0 and 1, respectively. Feature selection proceeded through a systematic multi-step validation process. Initially, interobserver reproducibility was assessed for all extracted radiomic features using the concordance correlation coefficient (CCC), with a stringent threshold of 0.85 applied to ensure robust feature stability. The subset of reproducible features underwent statistical evaluation using Wilcoxon rank-sum (WRS) tests, where features demonstrating significant discriminative capability (P < 0.1) were identified. Final feature refinement employed minimum redundancy maximum relevance (mRMR) analysis to optimize the feature set while controlling for multicollinearity, resulting in the selection of 20 CT-based radiomics features. To validate the methodological approach, principal component analysis (PCA) was conducted on both the post-WRS significant features and the mRMR-selected subset for comparative assessment of dimensionality reduction effectiveness and feature independence validation, thereby establishing the methodological integrity of the radiomics signature.

### Data partition and sampling strategy

The final dataset, comprising selected clinical predictors and radiomics features for each patient, was partitioned into training and testing cohorts through stratified randomization at a 7:3 ratio to maintain proportional RBP representation. Given the inherent class imbalance in the training cohort, a sequential sampling protocol was implemented to optimize the training data distribution. This protocol comprised adaptive synthetic oversampling (ADASYN) to generate synthetic RBP cases with emphasis on boundary regions 21, followed by TomekLinks undersampling to refine the control group distribution 22, ultimately achieving a more balanced representation for subsequent model development.

### Model training and hyperparameter optimization

Five AI prediction models were selected for RBP prediction: four machine learning (ML) algorithms including logistic regression (LR), which offered clear interpretability of feature relationships; random forest (RF) and XGBoost, which enabled identification of complex variable interactions and feature importance analysis; and support vector machine (SVM), which showed effectiveness in analyzing high-dimensional radiomics features. Additionally, TabNet, a deep learning (DL) architecture specifically optimized for tabular data, was implemented to leverage its sequential attention mechanism and dynamic feature selection capabilities.

For the ML models, hyperparameter optimization was conducted through randomized grid search with 5-fold cross-validation. The parameter grid for LR encompassed regularization strength and penalty type; RF optimization focused on tree depth, number of estimators, and minimum samples per leaf; XGBoost parameters included learning rate, maximum depth, and number of estimators; and SVM optimization addressed kernel selection, regularization parameter, and kernel coefficient. Each model underwent 100 iterations of parameter search, with area under the receiver operating characteristic curve (AUROC) serving as the optimization metric. Following the identification of optimal parameters, final models were trained on the complete training dataset using these optimized configurations to maximize the utilization of available training data.

For TabNet optimization, a systematic two-phase approach was implemented. The initial phase employed randomized grid search with 5-fold cross-validation to optimize architectural parameters, including feature transformation dimensions, decision steps, independent and shared feature layers, and sparsity coefficient. Each parameter combination was evaluated across 100 iterations, using AUROC as the optimization metric. During the training process, early stopping was implemented with a patience of 10 epochs on the validation set to prevent overfitting. The second phase focused on training parameters, optimizing learning rate, batch size, and decay schedule. After determining the optimal configuration, the final TabNet model was trained on the complete training dataset with a held-out validation set (ratio 8:2) for performance monitoring and early stopping.

### Model comparison

A model comparative test was conducted in the independent testing cohort through three critical dimensions to identify the optimal AI model for RBP risk stratification. First, discrimination analysis was conducted to assess each model's ability to differentiate between RBP and non-RBP cases. Second, calibration analysis examined the agreement between predicted probabilities and actual outcomes. Third, clinical utility was evaluated through the assessment of model prediction accuracy across different risk thresholds. Performance metrics derived from confusion matrices provided standardized evaluation parameters, including predictive values and classification accuracy.

### Interpretation of the optimal AI model

A systematic evaluation was conducted to examine the predictive mechanisms within the optimal AI model. Feature importance analysis identified and ranked the ten most influential variables for RBP prediction, incorporating both radiomics parameters and clinical indicators. An ablation experiment was subsequently performed to validate the predictive contribution of key features through sequential feature elimination, demonstrating their relative importance within the model framework.

### Statistical analysis

All statistical evaluations were performed using Python (version 3.12.0) with established analytical libraries. Demographic and clinical characteristics between RBP and control groups were compared using chi-square tests for categorical variables and WRS tests for continuous variables. Model performance assessment encompassed multiple complementary approaches: discrimination capability was quantified through ROC curve analysis and corresponding AUC values; calibration assessment utilized calibration curves complemented by Brier scores (BS) to evaluate prediction accuracy; and clinical utility was determined through decision curve analysis (DCA) to assess net benefit across different threshold probabilities. Comprehensive performance metrics were calculated from confusion matrices, including accuracy, precision, recall, F1-score, and Cohen's kappa coefficient, providing a thorough evaluation of classification performance.

## Results

### Patient characteristics and cohort distribution

**Figure 2:**
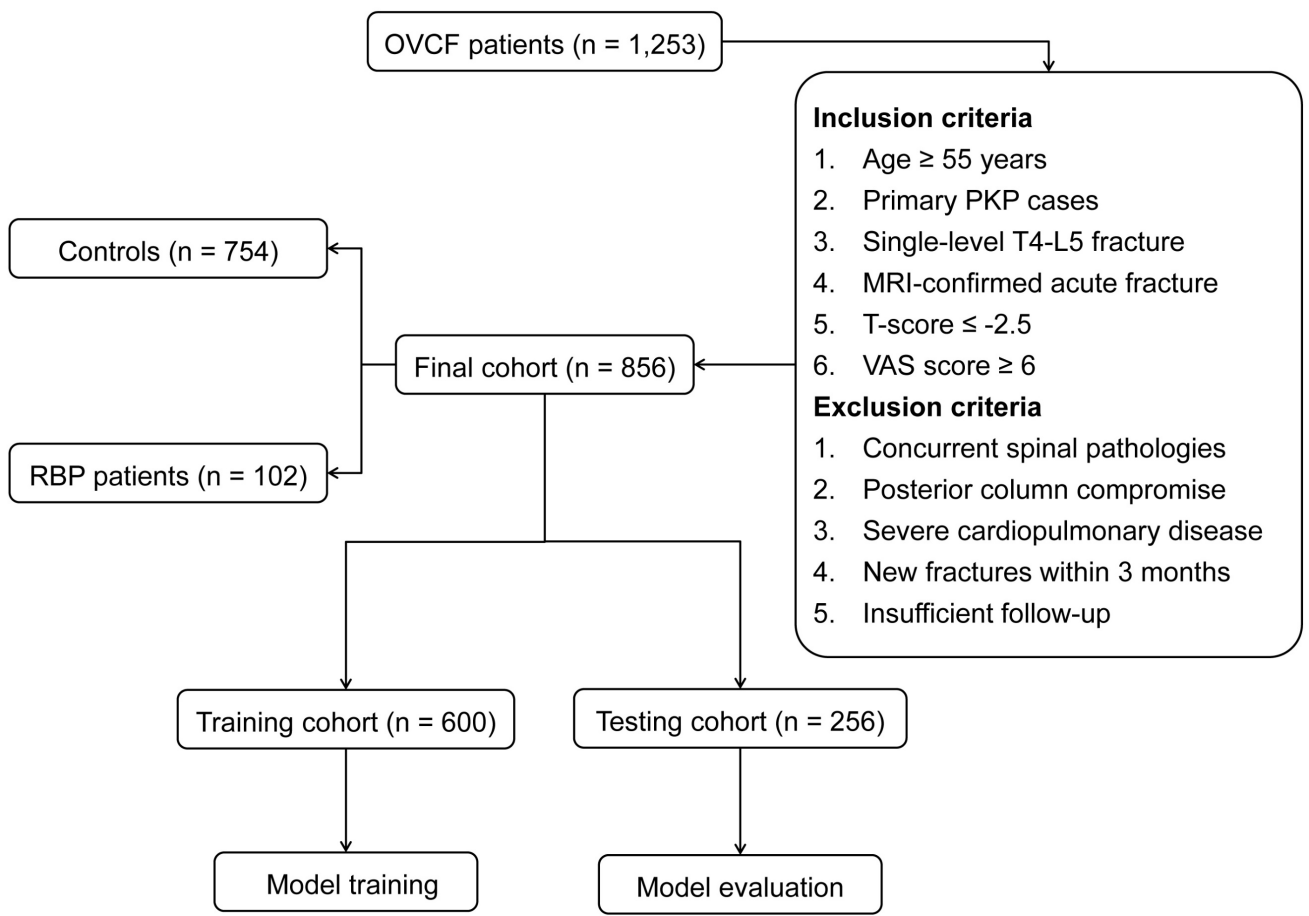
Flow chart illustrating patient selection and cohort distribution.

Among 1,253 OVCF patients initially screened, 856 eligible cases were enrolled after applying the inclusion and exclusion criteria (**Figure 2**). Of these enrolled patients, 102 (11.9%) developed RBP following VA procedures, while 754 patients maintained satisfactory outcomes. The study population was systematically allocated into training (n=600) and testing (n=256) cohorts, comprising 72 (12.0%) and 30 (11.7%) RBP patients, respectively. Comprehensive analysis of demographic and clinical characteristics revealed no statistically significant differences between the training and testing cohorts across all evaluated parameters (all *P* > 0.05), as detailed in **Table S1**.

### Clinical predictors of RBP

In the training cohort, several potential clinical predictors were identified through comparative analysis to be associated with RBP development. Lower BMD values and elevated preoperative ODI scores were observed in patients with RBP. The distribution of fracture locations was found to be significantly different, with T11-L2 fractures being predominantly observed in the RBP group. Moreover, imaging features including IVC and TLF injury were detected more frequently in patients who developed RBP (**Table 1**).

### Radiomics feature extraction and selection

**Figure 3:**
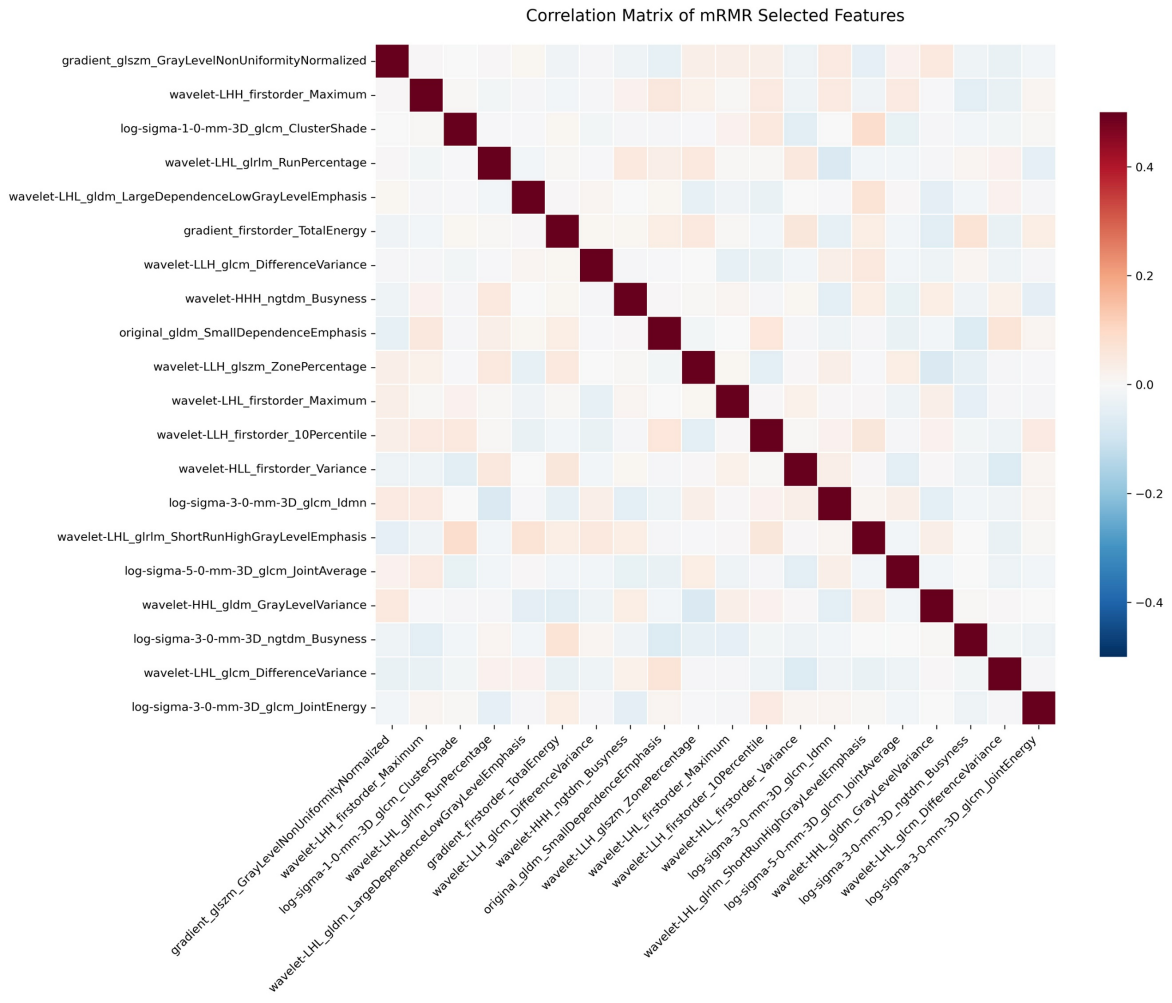
Correlation matrix of selected radiomics features. Heatmap visualization of correlations among 20 mRMR-selected features. Color intensity indicates correlation strength (red: positive correlation; blue: minimal correlation). The predominant light coloring demonstrates low inter-feature redundancy.

Initial radiomics analysis extracted 1,223 quantitative features from each segmented vertebral body. After standardization procedures, interobserver reproducibility evaluation retained 940 features demonstrating high stability (CCC ≥ 0.85). Subsequent statistical analysis identified 106 features with significant discriminative potential between RBP and control groups. The mRMR algorithm further refined this feature set to 20 optimal parameters (**Figure 3**). These selected features represented diverse aspects of vertebral characteristics, including intensity distribution metrics derived from first-order statistics, textural features obtained through gray-level matrices analysis, and advanced wavelet-based parameters capturing multi-scale image properties.

**Figure 4:**
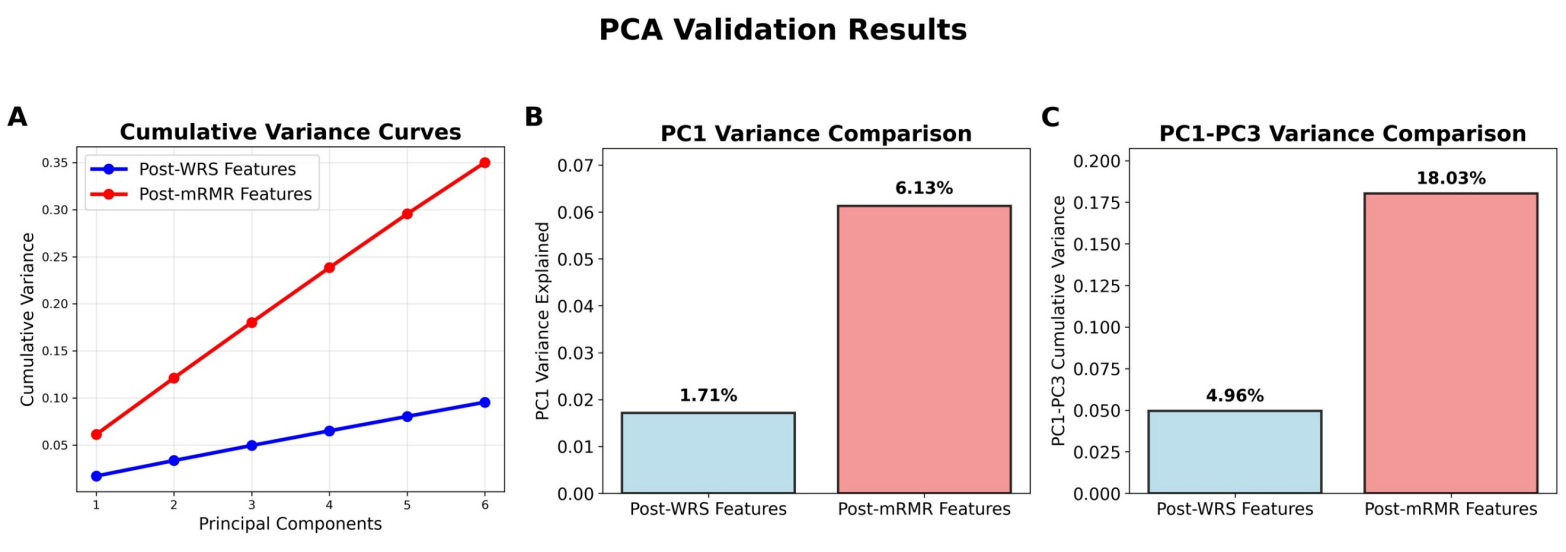
PCA validation results comparing feature independence between post-WRS and post-mRMR feature sets. (A) PC1 variance comparison. (B) PC1-PC3 cumulative variance comparison. (C) Cumulative variance curves across six principal components.

PCA of the post-WRS significant features revealed highly distributed variance across components, with the first principal component explaining only 1.7% of total variance and the first three components cumulatively accounting for 4.9% (**Figure 4A**). This uniform variance distribution indicated substantial feature diversity without dominant underlying patterns, supporting the rationale for the mRMR feature selection. Comparative PCA analysis of the mRMR-selected features demonstrated marked improvement in feature coherence, with the first principal component explaining 6.1% of variance (3.6-fold increase, **Figure 4B**) and the first three components cumulatively accounting for 18.0% (3.7-fold increase, **Figure 4C**). This enhanced variance concentration confirmed that mRMR successfully identified radiomics features that more effectively capture the underlying vertebral characteristics relevant to RBP prediction.

### Model training and optimization

Five predictive models were constructed to assess the risk of RBP, incorporating four ML approaches (LR, SVM, RF, and XGBoost) alongside a DL framework (TabNet). A systematic cross-validation strategy was implemented for parameter optimization, with hyperparameter combinations being systematically evaluated through 100 optimization cycles. The finalized hyperparameter configurations are documented in **Table S2**, which were subsequently employed for final model training on the complete training dataset.

### Model comparison and performance analysis

**Figure 5:**
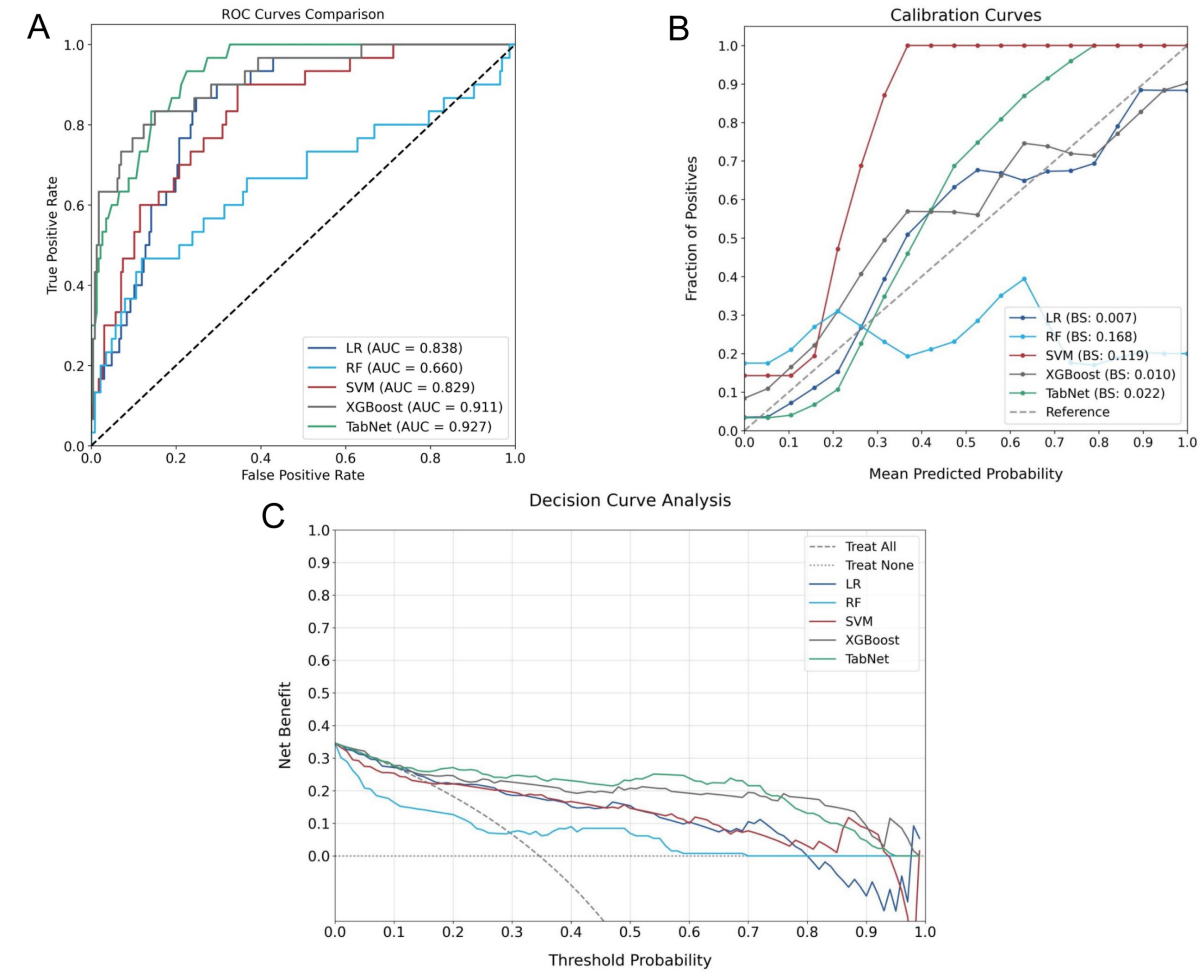
Performance evaluation of different AI Models for RBP prediction. A: ROC curves comparing discriminative capabilities. B: Calibration curves demonstrating probability estimation accuracy. C: DCA showing clinical utility across threshold probabilities.

**Figure 6:**
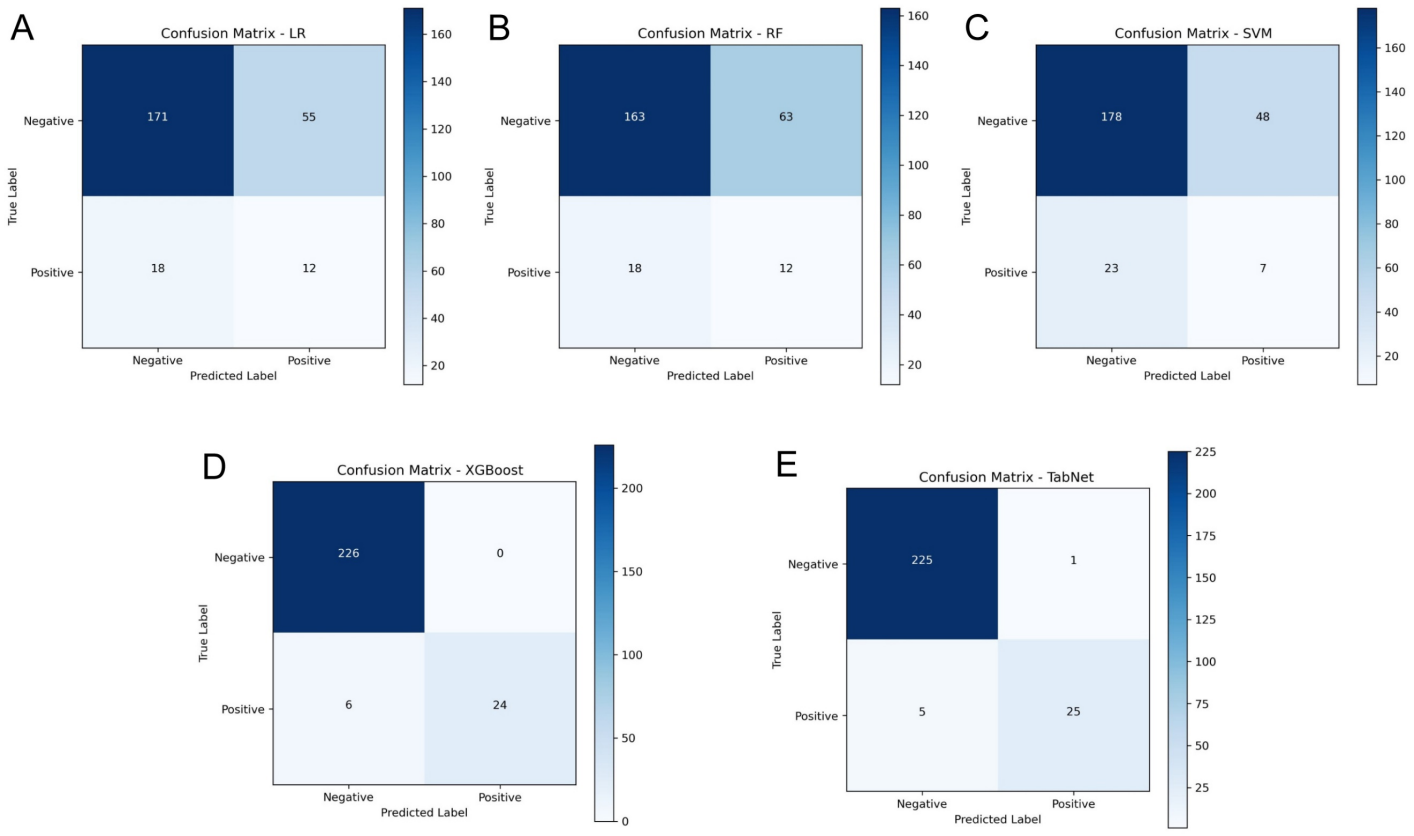
Confusion matrices of AI models in the independent testing cohort. A-E: Confusion matrices for LR, RF, SVM, XGBoost, and TabNet models, respectively.

A comparative experiment across multiple AI models demonstrated heterogeneous predictive capabilities, as illustrated in **Figure 5**, which provides a comprehensive evaluation through discrimination ability, calibration performance, and clinical utility assessment. In the testing cohort, TabNet and XGBoost were found to outperform others, with AUROC values of 0.927 and 0.911 being achieved, respectively. Model calibration comparison through BS values indicated higher probability estimation precision for these advanced models (TabNet: 0.022; XGBoost: 0.010), which reflected alignment between predicted and observed outcomes. Clinical utility of TabNet and XGBoost was further validated through DCA, where these models demonstrated sustained net benefit across threshold probabilities (0-95%). The confusion matrices (**Figure 6**) and performance metrics analysis (**Table 2**) revealed TabNet to demonstrate superior diagnostic capability, particularly in reducing false-negative predictions. Within the comparative framework, TabNet exhibited optimal sensitivity with a recall rate of 0.833, which enabled identification of one additional RBP case compared to XGBoost in the testing cohort, while equivalent accuracy (0.977) was maintained between these models. Based on the enhanced detection capability and robust performance metrics (F1 score: 0.893; Kappa: 0.882), TabNet was established as the optimal algorithmic framework for preoperative RBP risk stratification.

### Feature importance and ablation analysis

**Figure 7:**
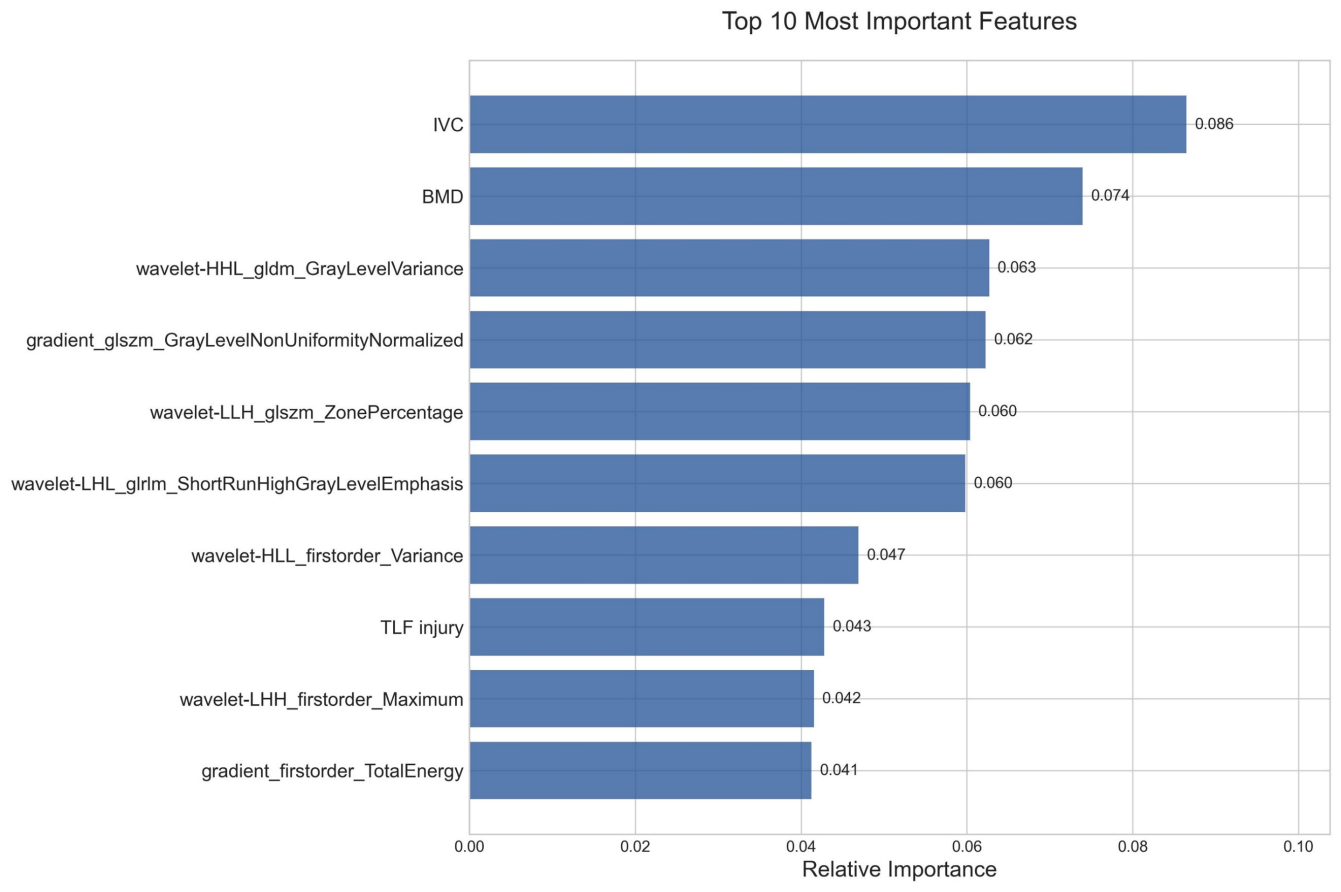
Relative importance of the top 10 features identified by the TabNet model in RBP prediction.

Feature importance analysis through the TabNet model identified ten critical predictive variables, as shown in **Figure 7**. Among these, IVC and BMD were determined as the primary clinical predictors, indicating the significance of structural defects and bone quality in RBP development. The remaining seven radiomics features were predominantly characterized by wavelet-based parameters, comprising two distinct categories: texture features that captured tissue heterogeneity patterns, and quantitative intensity metrics that reflected vertebral density distribution. These complementary radiomics characteristics, quantifying both structural heterogeneity and density variations, demonstrated significant predictive value for RBP development.

The contribution patterns were systematically validated through two-phase ablation experiments, with results presented in **Table 3**. In the first phase, sequential elimination of the top five features demonstrated a graduated impact on model performance, where the removal of higher-ranked features resulted in more substantial performance decreases. In the second phase, complete ablation of radiomics features led to a marked reduction in true negative predictions (from 225 to 157), indicating compromised specificity in identifying non-RBP cases. Conversely, the elimination of clinical features resulted in a significant increase in false positives (from 1 to 137), suggesting that clinical parameters were instrumental in reducing overdiagnosis. These findings indicated that while radiomics features enhanced the model's ability to correctly identify non-RBP cases, clinical parameters were crucial for maintaining diagnostic precision.

## Discussion

Despite the widespread implementation of VA procedures for OVCFs, the persistent challenge of RBP continues to impact patient outcomes, with current prediction models based on conventional clinical parameters showing limited preoperative risk stratification capabilities. In this study, we developed and validated an innovative AI framework that integrates CT-based radiomics features with clinical parameters for preoperative RBP prediction, achieving remarkable predictive performance through the TabNet architecture. This comprehensive model demonstrates superior capability in identifying patients at risk for RBP compared to other AI models, with its feature importance analysis revealing novel insights into the predictive value of wavelet-based texture parameters and quantitative intensity metrics alongside established clinical risk factors. Our study represents the first systematic comparison of multiple radiomics-based AI algorithms for RBP prediction in OVCF patients, establishing a robust framework that not only enhances preoperative risk stratification but also provides clinically actionable insights for personalized treatment planning, potentially enabling early preventive interventions for high-risk patients.

CT-based radiomics offers unique advantages in analyzing vertebral pathology, primarily due to its superior capability in providing comprehensive three-dimensional visualization of the injured vertebrae with high spatial resolution and excellent bone-soft tissue contrast 23. This imaging modality enables detailed characterization of both cortical and trabecular bone architecture, allowing for extraction of quantitative features that capture subtle structural variations that may be imperceptible to visual assessment 24. The emerging field of CT-based radiomics in vertebral analysis has gained increasing attention in recent years, with several pioneering studies demonstrating its potential utility. Previous investigations focusing on fracture characterization have consistently identified gray-level textural features and intensity-based statistical parameters as key predictors 13, 25. Our analysis of RBP prediction revealed similar patterns but demonstrated a critical finding regarding the predominance of wavelet-transformed features among our top predictors. Notably, five of the seven radiomics features required wavelet transformation to achieve predictive value, including three texture-based features and two first-order statistical features. This pattern indicates that the microstructural alterations associated with RBP are not evident in raw CT images but emerge only when vertebral structure is analyzed at specific spatial frequencies. The texture features likely remained non-predictive in their original form because normal trabecular patterns mask subtle pathological changes. In contrast, wavelet decomposition successfully isolates these abnormalities by separating high-frequency components that reveal microfractures from low-frequency components that capture architectural deformities 26. Similarly, first-order intensity features become informative only after wavelet filtering separates pathological variations from the inherent density heterogeneity of osteoporotic bone. This frequency-specific information suggests that RBP development involves multi-scale structural abnormalities ranging from fine trabecular disruptions to larger architectural distortions affecting load distribution. The emergence of gradient-based features as additional predictors further supports this interpretation by identifying transition zones representing active remodeling or incomplete healing 27. When compared to our preliminary research utilizing traditional linear prediction methods 15, the current AI-based approach revealed a more diverse feature set. This expanded feature profile likely reflects the TabNet architecture's superior capability in capturing complex non-linear relationships within the imaging data, particularly through its attention mechanism that dynamically weights different frequency components based on their predictive relevance 28.

In the comparative analysis of AI models, TabNet and XGBoost demonstrated superior predictive capabilities, primarily due to their optimized architectures for processing tabular data with complex feature interactions 29, 30. TabNet exhibited enhanced sensitivity compared to other models, aligning with clinical priorities wherein identification of potential RBP cases takes precedence over false-positive reduction. This characteristic, combined with robust overall performance metrics, established TabNet as the optimal model for clinical implementation. Feature importance analysis indicated that radiomics features constituted seven of the top ten predictive variables, with clinical parameters including IVC, BMD, and TLF injury comprising the remainder. Although IVC and BMD demonstrated the highest individual predictive weights among all variables, the collective contribution of radiomics features was determined to be substantial for model performance. Ablation experiments substantiated this observation, wherein exclusion of radiomics features resulted in marked deterioration in model precision, indicating their essential role in reducing false-positive predictions. The model performance declined more significantly when clinical features were removed, suggesting that accurate RBP risk stratification requires integration of both radiomics and clinical parameters. These findings demonstrate the complementary roles of clinical variables in providing fundamental risk assessment and radiomics features in enhancing discriminative capability through tissue characterization.

The developed predictive framework advances the field of RBP prediction through its novel integration of CT-based radiomics features with AI algorithms. To our knowledge, this represents the first investigation to utilize quantitative imaging biomarkers for preoperative RBP risk assessment in OVCF patients, whereas previous studies relied exclusively on clinical parameters analyzed through traditional statistical methods 19, 20, 31. Compared to our preliminary radiomics-based nomogram study 15, the current investigation demonstrates substantial methodological improvements through the transition from linear regression to sophisticated AI algorithms capable of capturing complex non-linear relationships, implementation of rigorous multi-step feature selection, and incorporation of an independent testing cohort to ensure model generalizability. These advances enabled the identification of previously unrecognized predictive patterns, resulting in significantly enhanced predictive performance that surpasses both our preliminary work and existing clinical prediction models. This enhanced discrimination capability enables accurate preoperative identification of patients at elevated risk for RBP, facilitating the implementation of individualized preventive strategies and patient-specific treatment planning. Furthermore, the model provides an objective, evidence-based tool for physicians to conduct informed discussions with patients regarding expected outcomes and potential complications, thereby establishing realistic therapeutic expectations. By transforming RBP management from reactive treatment to proactive prevention through personalized risk-adapted care pathways, this AI-driven radiomics approach offers significant potential for improving surgical outcomes and patient satisfaction in vertebral augmentation procedures.

However, several limitations warrant consideration in interpreting these findings. First, the study population was derived from a single medical center, potentially limiting the generalizability of results across different clinical settings and patient populations. Second, as a preoperative prediction model, our approach was intentionally restricted to preoperative parameters to enable early risk stratification. However, this design inherently excludes potentially significant intraoperative and postoperative factors, such as cement injection pressure, cement viscosity, and cement distribution patterns, that may substantially influence RBP development and treatment outcomes 32. Third, the model application remained restricted to single-segment vertebral fractures, and validation in patients with multiple OVCF segments has yet to be conducted. Future investigations should focus on developing dynamic prediction models that can incorporate real-time perioperative variables to adjust initial risk predictions, thereby providing more clinically relevant and adaptive risk stratification tools. Additionally, multicenter validation studies and model adaptation for complex vertebral fracture patterns will facilitate broader clinical implementation and expand the applications of RBP risk prediction in VA procedures.

In conclusion, an innovative TabNet DL framework integrating CT-based radiomics features with clinical parameters was established for predicting RBP. The model demonstrated robust predictive performance through the synergistic contribution of clinical and radiomics features. This predictive tool enables preoperative risk stratification and facilitates personalized treatment planning in VA procedures.

## Supplementary Material

Supplementary tables.

## Figures and Tables

**Table 1:** Comparison of clinical characteristics between control and RBP groups in the training cohort

Variable		Control group (n=528)	RBP group (n=72)	P value
Age, year		70 (67, 72)	69 (67, 73)	0.249
BMI, kg/m^2^		23.5 (21.8, 25.4)	23.7 (22.1, 25.4)	0.896
BMD, T-score		-3.1 (-3.3, -2.8)	-3.3 (-3.6, -3.0)	<0.001
Preoperative VAS, score		7 (6, 8)	7 (6, 8)	0.298
Preoperative ODI, score		42 (40, 44)	43 (41, 45)	0.041
Vertebral height loss (%)		33.9 (30.1, 37.6)	33.4 (28.7, 36.3)	0.161
Cobb angle, (°)		27.1 (24.0, 30.1)	27.0 (23.3, 29.4)	0.173
Gender, n(%)	Male	109 (20.6)	19 (26.4)	0.264
	Female	419 (79.4)	53 (73.6)	
Fracture position, n (%)	T4 - T10	51 (9.7)	7 (9.7)	0.009
	T11 - L2	249 (47.2)	47 (65.3)	
	L3 - L5	228 (43.2)	18 (25.0)	
Hypertension, n (%)		233 (44.1)	30 (41.7)	0.693
Diabetes, n (%)		49 (9.3)	6 (8.3)	0.794
Smoking, n (%)		86 (16.3)	9 (12.5)	0.409
IVC, n (%)		39 (7.4)	11 (15.3)	0.023
TLF injury, n (%)		31 (5.9)	13 (18.1)	<0.001

**Table 2:** Comparative performance metrics of AI Models in RBP prediction

Model	Accuracy	Precision	Recall	F1 score	Kappa
LR	0.715	0.179	0.400	0.247	0.104
RF	0.684	0.160	0.400	0.229	0.073
SVM	0.723	0.127	0.233	0.164	0.014
XGBoost	0.977	1.000	0.800	0.889	0.878
TabNet	0.977	0.962	0.833	0.893	0.882

**Table 3:** Ablation analysis of feature contributions to model performance in RBP prediction.

Model	TP	TN	FP	FN	Accuracy	Precision	Recall	F1 score	Kappa
Baseline model	25	225	1	5	0.977	0.962	0.833	0.893	0.882
Without IVC	8	204	22	22	0.828	0.267	0.267	0.267	0.265
Without BMD	11	211	15	19	0.867	0.423	0.367	0.393	0.330
Without wavelet-HHL_gldm_GrayLevelVariance	15	219	7	15	0.914	0.682	0.500	0.577	0.520
Without gradient_glszm_GrayLevelNonUniformityNormalized	13	223	3	17	0.922	0.813	0.433	0.565	0.527
Without wavelet-LLH_glszm_ZonePercentage	20	218	8	10	0.930	0.714	0.667	0.690	0.668
Without clinical features	20	89	137	10	0.426	0.127	0.667	0.214	0.021
Without radiomics features	22	157	69	8	0.699	0.242	0.733	0.364	0.120

TP: true positive; TN: true negative; FP: false positive; FN: false negative

## References

[1] Patel D, Liu J, Ebraheim NA (2022). Managements of osteoporotic vertebral compression fractures: A narrative review. World journal of orthopedics.

[2] Parreira PCS, Maher CG, Megale RZ, March L, Ferreira ML (2017). An overview of clinical guidelines for the management of vertebral compression fracture: a systematic review. The Spine Journal.

[3] Long Y, Yi W, Yang D (2020). Advances in Vertebral Augmentation Systems for Osteoporotic Vertebral Compression Fractures. Pain Research and Management.

[4] Yang X-G, Dong Y-Q, Liu X, Liu X-L, Luo H-T, Bao Y (2024). Incidence and prognostic factors of residual back pain in patients treated for osteoporotic vertebral compression fractures: a systematic review and meta-analysis. European Spine Journal.

[5] Wang S, Shi M, Zhou X, Yu J, Han M, Zhang X (2025). Predicting residual pain after vertebral augmentation in vertebral compression fractures: a systematic review and critical appraisal of risk prediction models. BMC Musculoskeletal Disorders.

[6] Inose H, Kato T, Ichimura S, Nakamura H, Hoshino M, Togawa D (2021). Predictors of residual low back pain after acute osteoporotic compression fracture. Journal of Orthopaedic Science.

[7] Li Y, Yue J, Huang M, Lin J, Huang C, Chen J (2020). Risk factors for postoperative residual back pain after percutaneous kyphoplasty for osteoporotic vertebral compression fractures. European Spine Journal.

[8] Inose H, Kato T, Ichimura S, Nakamura H, Hoshino M, Takahashi S (2022). Factors Contributing to Residual Low Back Pain after Osteoporotic Vertebral Fractures. Journal of Clinical Medicine.

[9] Jiang T, Lau S-H, Zhang J, Chan L-C, Wang W, Chan P-K (2024). Radiomics signature of osteoarthritis: Current status and perspective. Journal of Orthopaedic Translation.

[10] Koch KM, Potter HG, Koff MF (2025). MRI-based radiomic analysis of soft tissue reactions near total hip arthroplasty. Journal of Orthopaedic Research.

[11] Mackin D, Fave X, Zhang L, Fried D, Yang J, Taylor B (2015). Measuring Computed Tomography Scanner Variability of Radiomics Features. Investigative Radiology.

[12] Duan S, Hua Y, Cao G, Hu J, Cui W, Zhang D (2023). Differential diagnosis of benign and malignant vertebral compression fractures: Comparison and correlation of radiomics and deep learning frameworks based on spinal CT and clinical characteristics. European Journal of Radiology.

[13] Kim AY, Yoon MA, Ham SJ, Cho YC, Ko Y, Park B (2022). Prediction of the Acuity of Vertebral Compression Fractures on CT Using Radiologic and Radiomic Features. Academic Radiology.

[14] Wang X, Ye W, Gu Y, Gao Y, Wang H, Zhou Y (2025). Predicting Secondary Vertebral Compression Fracture After Vertebral Augmentation via CT-Based Machine Learning Radiomics-Clinical Model. Academic Radiology.

[15] Ge C, Chen Z, Lin Y, Zheng Y, Cao P, Chen X (2022). Preoperative prediction of residual back pain after vertebral augmentation for osteoporotic vertebral compression fractures: Initial application of a radiomics score based nomogram. Frontiers in Endocrinology.

[16] Koçak B, Durmaz E, Ateş E, Kılıçkesmez Ö (2019). Radiomics with artificial intelligence: a practical guide for beginners. Diagnostic and interventional radiology (Ankara, Turkey).

[17] Sollini M, Antunovic L, Chiti A, Kirienko M (2019). Towards clinical application of image mining: a systematic review on artificial intelligence and radiomics. European Journal of Nuclear Medicine and Molecular Imaging.

[18] Wang Z-W, Wang G-Y, Liu D-K, Zhang D-Z, Zhao C (2023). Risk Factors for Residual Back Pain After PVP Treatment for osteoporotic Thoracolumbar Compression Fractures: A Retrospective Cohort Study. World Neurosurgery.

[19] Li Q, Shi L, Wang Y, Guan T, Jiang X, Guo D (2021). A Nomogram for Predicting the Residual Back Pain after Percutaneous Vertebroplasty for Osteoporotic Vertebral Compression Fractures. Pain Research and Management.

[20] Wu H, Li C, Song J, Zhou J (2024). Developing predictive models for residual back pain after percutaneous vertebral augmentation treatment for osteoporotic thoracolumbar compression fractures based on machine learning technique. Journal of Orthopaedic Surgery and Research.

[21] Haibo H, Yang B, Garcia EA, Shutao L (2008). ADASYN: Adaptive synthetic sampling approach for imbalanced learning. 2008 IEEE International Joint Conference on Neural Networks (IEEE World Congress on Computational Intelligence).

[22] Yang G, Wang G, Wan L, Wang X, He Y (2025). Utilizing SMOTE-TomekLink and machine learning to construct a predictive model for elderly medical and daily care services demand. Sci Rep.

[23] Chen B, Cui J, Li C, Xu P, Xu G, Jiang J (2024). Application of radiomics model based on lumbar computed tomography in diagnosis of elderly osteoporosis. Journal of Orthopaedic Research.

[24] Fritz B, Yi PH, Kijowski R, Fritz J (2023). Radiomics and Deep Learning for Disease Detection in Musculoskeletal Radiology: An Overview of Novel MRI- and CT-Based Approaches. Invest Radiol.

[25] Li W-G, Zeng R, Lu Y, Li W-X, Wang T-T, Lin H (2023). The value of radiomics-based CT combined with machine learning in the diagnosis of occult vertebral fractures. BMC Musculoskeletal Disorders.

[26] Abid Fourati W, Bouhlel MS (2014). Trabecular Bone Image Segmentation Using Wavelet and Marker-Controlled Watershed Transformation. Chinese Journal of Engineering.

[27] Ren T, Klein K, von Rechenberg B, Darwiche S, Dailey HL (2022). Image-based radiodensity profilometry measures early remodeling at the bone-callus interface in sheep. Biomech Model Mechanobiol.

[28] Fan Y, Waldmann P (2024). Tabular deep learning: a comparative study applied to multi-task genome-wide prediction. BMC Bioinformatics.

[29] Zhang Z, Pan Y, Lu Y, Ye L, Zheng M, Zhang G (2024). The TabNet Model for Diagnosing Axial Spondyloarthritis Using MRI Imaging Findings and Clinical Risk Factors. Int J Rheum Dis.

[30] Dong J, Peng L, Yang X, Zhang Z, Zhang P (2022). XGBoost-based intelligence yield prediction and reaction factors analysis of amination reaction. J Comput Chem.

[31] Liu Z, Zhang X, Liu H, Wang D (2022). A nomogram for short-term recurrent pain after percutaneous vertebroplasty for osteoporotic vertebral compression fractures. Osteoporosis International.

[32] Yang JS, Liu JJ, Chu L, Li J, Chen C, Chen H (2019). Causes of Residual Back Pain at Early Stage After Percutaneous Vertebroplasty: A Retrospective Analysis of 1,316 Cases. Pain physician.

