# Supplementary material for: Comparison of Artificial Intelligence Models Using CT Radiomics for Predicting Post-Vertebral Augmentation Residual Back Pain in Osteoporotic Vertebral Compression Fractures
Source: Int J Med Sci. 2025 Jul 11;22(13):3329–41. doi: 10.7150/ijms.114419 (PMC12320780; doi:10.7150/ijms.114419)
Supplement: Supplementary file 1 — Supplementary tables. [file ijmsv22p3329s1.pdf]

Supplementary

Table S1. Baseline characteristics of the training and testing cohorts

Variable		Training cohort (n=600)	Testing cohort (n=256)	*P* value
Age, year		70 (67, 72)	70 (66, 73)	0.643
BMI, kg/m^2		23.7 (21.8, 25.6)	24.1 (22.4, 25.9)	0.230
BMD, T-score		-3.1 (-3.4, -2.9)	-3.0 (-3.2, -2.8)	0.291
Preoperative VAS, score		7 (6, 8)	7 (6, 8)	0.950
Preoperative ODI, score		42 (40, 44)	42 (39, 44)	0.952
Vertebral height loss (%)		33.9 (29.9, 37.4)	34.5 (30.3, 38.3)	0.241
Cobb angle, (°)		27.1 (23.8, 30.0)	27.6 (24.2, 30.9)	0.228
Gender, n (%)	Male	128 (21.3)	59 (23.0)	0.579
	Female	472 (78.7)	197 (77.0)	
Fracture position, n (%)	T4 - T10	58 (9.7)	28 (10.9)	0.487
	T11 - L2	296 (49.3)	115 (44.9)	
	L3 - L5	246 (41.0)	113 (44.1)	
Hypertension, n (%)		263 (43.8)	116 (45.3)	0.690
Diabetes, n (%)		55 (9.2)	25 (9.8)	0.783
Smoking, n (%)		95 (15.8)	45 (17.6)	0.527
IVC, n (%)		50 (8.3)	26 (10.2)	0.391

TLF injury, n (%)	44 (7.3)	12 (4.7)	0.152
RBP presence, n (%)	72 (12.0)	30 (11.7)	0.907

Table S2. Optimal hyperparameter configurations of AI models for RBP risk prediction

Model	Parameter	Values
LR	C	0.001
	Penalty	l2
	Solver	saga
	Tolerance (tol)	1e-4
	random_state	42
RF	n_estimators	300
	max_features	sqrt
	max_depth	None
	min_samples_split	4
	min_samples_leaf	2
SVM	C	1.0
	Kernel	rbf
	Gamma	scale
	random_state	42

XGBoost	n_estimators	100
	learning_rate	0.1
	max_depth	3
	min_child_weight	3
	Gamma	0
	Subsample	0.9
TabNet	Decision Dimension (n_d)	8
	Attention Dimension (n_a)	20
	Decision Steps (n_steps)	5
	Gamma	1.0
	Seed	42
	learning_rate	0.01

C: Regularization parameter, l2: Ridge regularization, saga: Stochastic average gradient with acceleration, Gamma: Minimum loss reduction,
